# *Notes from the Field*: Tetanus in Four Children — Idaho, Minnesota, Missouri, and Wisconsin, 2024

**DOI:** 10.15585/mmwr.mm7514a2

**Published:** 2026-04-16

**Authors:** Katherine L. Campbell, Avnika B. Amin, Jessica Goswitz, Rebecca Betz, Stacey Moyer, Jayne Griffith, Kris K. Carter, Christine Hahn, Michelle M. Hughes

**Affiliations:** ^1^Idaho Department of Health and Welfare; ^2^Epidemic Intelligence Service, CDC; ^3^Division of Bacterial Diseases, National Center for Immunization and Respiratory Diseases, CDC; ^4^Missouri Department of Health and Senior Services; ^5^Panhandle Health District, Hayden, Idaho; ^6^Wisconsin Department of Health Services; ^7^Minnesota Department of Health; ^8^Division of State and Local Readiness, Office of Readiness and Response, CDC.

**Summary**

**What is already known about this topic?**

Because of high coverage with recommended tetanus toxoid–containing vaccine (TTCV), pediatric tetanus is rare in the United States; approximately four cases are reported annually.

**What is added by this report?**

Among four U.S. children who developed tetanus in 2024, none had completed a primary TTCV series, and none received TTCV or tetanus immunoglobulin (TIG) prophylaxis after their exposure and before illness onset. All four patients required hospitalization, ranging from 8 to 45 days, and two required additional rehabilitation care. Only one child completed the TTCV series after illness.

**What are the implications for public health practice?**

Completing a primary TTCV series and remaining up to date with TTCV vaccination are essential to preventing tetanus; patients with tetanus-prone wounds should receive timely administration of TTCV and TIG according to recommendations.

Tetanus is an acute neuromuscular disease mediated by a toxin produced by *Clostridium tetani* bacteria.* *C*. *tetani* spores are ubiquitous in the environment, including in soil, dust, and manure, and are often introduced into the body through an injury. *C*. *tetani* spores enter the body, germinate, and produce tetanospasmin, a potent neurotoxin that can cause severe, sometimes fatal, disease. Persons can help prevent tetanus by remaining up to date with recommended tetanus toxoid–containing vaccine (TTCV) and by receiving postwound prophylaxis for tetanus-prone wounds, including wound care and administration of TTCV or tetanus immunoglobulin (TIG); the treatment regimen is based on multiple clinical considerations (*1*). In the United States, pediatric tetanus is rare because of high coverage with recommended TTCV doses,^†^ although pediatric TTCV vaccination coverage varies by state (*1*,*2*). A recent surveillance summary reported that among persons with tetanus whose vaccination history was known, 44% had not received a TTCV dose (*1*). During 2013–2023, an average of 4.4 U.S. pediatric tetanus cases were identified each year in the CDC National Notifiable Diseases Surveillance System (NNDSS) (*1*). In July 2024, the first pediatric case of tetanus in Idaho in >30 years was reported. Three other U.S. pediatric cases were reported in three other states in 2024. An investigation was initiated to understand patient characteristics and the circumstances under which these cases occurred, and to guide prevention efforts.

## Investigation and Outcomes

### Data Source and Analysis

Four probable cases of tetanus among patients aged <18 years in Idaho, Minnesota, Missouri, and Wisconsin were identified through NNDSS. A descriptive analysis was performed, based on information obtained from case reports and medical chart abstraction; this information included patient age, TTCV-vaccination status, characteristics of the suspected implicated injury, clinical course, and subsequent receipt of TTCV vaccination. This activity was reviewed by CDC, deemed not research, and was conducted consistent with applicable federal law and CDC policy.^§^

### Patient Characteristics

Patient age groups ranged from <5 years to 10–15 years (Table). Two patients lived in metropolitan counties, and two lived in nonmetropolitan counties. At the time of exposure, no patient had received any vaccination against tetanus.

**TABLE:** Characteristics, vaccination status, and clinical course of four children aged 1–15 years with a tetanus diagnosis — Idaho, Minnesota, Missouri, and Wisconsin, 2024

Patient	Age group, yrs	Metro or nonmetro county*	TTCV status	Likely exposure route	Days from injury to illness	Sought care before illness	TIG and TTCV prophylaxis before illness	Days in hospital, TIG treatment and TTCV receipt^†^	Follow-up care after release
A	10–15	Metro	Unvaccinated	Compound ankle fracture from riding electric scooter	8	Yes, when injured	Offered by health care provider and declined by parents	8 days, received TIG and 1 dose TTCV	Yes
B	5–9	Nonmetro	Unvaccinated	Unknown	Unknown	No	Did not seek care before illness onset	31 days, received TIG and 2 doses TTCV	Unknown
C	1–4	Metro	Unvaccinated	Knee puncture from animal bone	10	Yes, 8 days after injury	Offered by health care provider and declined by parents	16 days, received TIG and 4 doses TTCV	Yes
D	10–15	Nonmetro	Unvaccinated	Crush foot injury from horse hoof while barefoot	7	No	Did not seek care before illness onset	45 days, received TIG and 2 doses TTCV	Unknown

**Abbreviations**: metro = metropolitan; nonmetro = nonmetropolitan; TIG = tetanus immunoglobulin; TTCV = tetanus toxoid–containing vaccine.

* County of patient’s residence. Metro versus nonmetro classification determined using the National Center for Health Statistics Urban-Rural Classification Scheme for Counties.

^†^ All patients received first TTCV doses in the hospital for prevention of future tetanus. A diagnosis of tetanus does not confer immunity against future disease; persons who have had tetanus disease need to complete a TTCV series to be protected against future tetanus.

### Exposure Route

Three patients with a hypothesized route of exposure had sustained their injury 7–10 days before symptom onset. Likely routes of exposure included 1) an ankle fracture (including traumatic injury to the overlying skin) during outdoor recreation, 2) a foot injury from a horse hoof while the child was barefoot, and 3) a knee puncture wound from an animal bone. The exposure mechanism for the fourth patient was unknown. Two of the patients (aged 5–9 and 10–15 years) did not seek medical care between the time the injury was sustained and the onset of tetanus. Two patients (aged <5 and 10–15 years) who did seek medical care were offered TTCV and TIG prophylaxis; however, in both cases, the parents declined prophylactic treatment.

### Tetanus Disease and Hospital Course

All four patients experienced generalized tetanus. Common symptoms included back, neck, and jaw pain; muscle spasms and muscle rigidity; and difficulty walking. All patients were hospitalized (mean duration = 25 days; range = 8–45 days), and all received TIG for treatment and an initial TTCV dose for prevention of future disease. Two patients had documentation of receipt of a second TTCV dose; only one patient subsequently completed the recommended primary TTCV vaccination series. At least two patients received postdischarge clinical care, including readmission for inpatient rehabilitation. No deaths occurred.

## Preliminary Conclusions and Actions

Tetanus can result in serious health consequences requiring extensive and costly medical care (*3*). Missed prevention opportunities for the children described in this report included failure to be vaccinated before the injury, delays in wound care, and lack of timely administration of TIG after exposure and before illness onset (*3*–*5*), including refusal. Tetanus disease is not transmitted person-to-person; therefore, herd immunity is not a feasible prevention strategy, nor does infection confer natural immunity: administration of TTCV is needed to prevent reinfection. Health care providers should discuss with parents the importance of being up to date with all recommended vaccines, including TTCV, and highlight the need for early medical care after a potentially contaminated wound occurs.^¶^ The need for prompt wound care is especially important in the case of environmentally contaminated or penetrating wounds; administration of TIG or TTCV when indicated should not be delayed, especially in unvaccinated or undervaccinated children.

## Footnotes

*
Chapter 16: Tetanus | Manual for the Surveillance of Vaccine-Preventable Diseases | CDC

† TTCV is usually administered to children as a 5-dose series of diphtheria and tetanus toxoids and acellular pertussis vaccine (DTaP) beginning at age 2 months.

§ 45 C.F.R. part 46.102(l)(2), 21 C.F.R. part 56; 42 U.S.C. Sect. 241(d); 5 U.S.C. Sect. 552a; 44 U.S.C. Sect. 3501 et seq.

¶ A wound contaminated with dirt, feces, soil, or saliva; puncture wounds; avulsions; and wounds resulting from missiles, crushing, burns, and frostbite.
